# Valgus knee deformity due to nonunion of lateral Hoffa fracture: A case report

**DOI:** 10.1016/j.tcr.2022.100662

**Published:** 2022-05-24

**Authors:** Masaki Iguchi, Tsuneari Takahashi, Mitsuharu Nakashima, Tomohiro Matsumura, Katsushi Takeshita

**Affiliations:** a Department of Orthopaedic Surgery, School of Medicine, Jichi Medical University, Shimotsuke, Japan; b Department of Orthopaedic Surgery, Ishibashi General Hospital, Shimotsuke, Japan; c Jichi Medical University Hospital Life Saving Emergency Center, Shimotsuke, Japan

**Keywords:** Hoffa fracture, Lateral femoral condyle, Nonunion, Internal fixation, Arthroscopy

## Abstract

Hoffa fractures are rare intra-articular injuries, and nonunion of Hoffa fractures is rarer. We report the case of an adult male with a nonunion of a Hoffa fracture by open reduction and internal fixation in which the lateral meniscus tear was treated by an arthroscopic surgery. A healthy 38-year-old man who had a history of untreated trauma to the left knee in a motorcycle accident 11 years ago presented to our hospital with the complaint of chronic left knee pain for 5 years. The patient had an obvious valgus knee with 0°–140° of motion, and radiographs revealed the nonunion of the left lateral Hoffa fracture (Letenneur type-III). Routine arthroscopic evaluation and a lateral meniscus posterior tear repair using all inside device were performed. The knee joint was exposed using a lateral para patella approach. The fracture was fixed with three 4.5-mm headless screws and distal femoral locking plates. Mobilization was started from the first operative day. Full weight bearing was allowed 8 weeks postoperatively. At the 1-year follow up, the X-ray showed healing of the nonunion site with no displacement of the Hoffa fracture. The knee range of motion, lower limb alignment, and clinical outcome were also improved. Nonunion of the Hoffa fracture should be treated by an internal fixation despite the chronicity.

## Introduction

Coronal fractures of the femoral condyle, also known as Hoffa fractures, are rare intra-articular injuries. Surgical treatment with open reduction and internal fixation is recommended for Hoffa fractures because conservative treatment is associated with a risk of displacement of the fracture fragment, nonunion, and avascular necrosis [1]. Nonunion of Hoffa fractures is rarer, and most studies on Hoffa fractures are case reports [1], [2]. Various case reports have suggested that cases of nonunion of Hoffa fractures should be treated with a debridement and open reduction and internal fixation. Headless screws, lateral LCP plates, combination of the lateral extra-articular buttress plates, and two cannulated lag screws are often used for the treatment of Hoffa fractures in patients with nonunions [1], [2], [3], [4]. This article presents the case of a 38-year-old man with a valgus knee deformity due to the nonunion of a Hoffa fracture that occurred 11 years ago and was treated with headless screws and LCP plates, resulting in a favorable clinical outcome and lower limb alignment.

## Case presentation

A healthy 38-year-old male presented to our hospital with the complaint of chronic left knee pain for 5 years with a history of untreated trauma to the left knee in a motorcycle accident 11 years ago. The patient was diagnosed with left Hoffa fracture and ipsilateral lower leg fracture. He was operated on the lower leg fracture but given conservative treatment for the Hoffa fracture at that time.

**Fig. 1:**
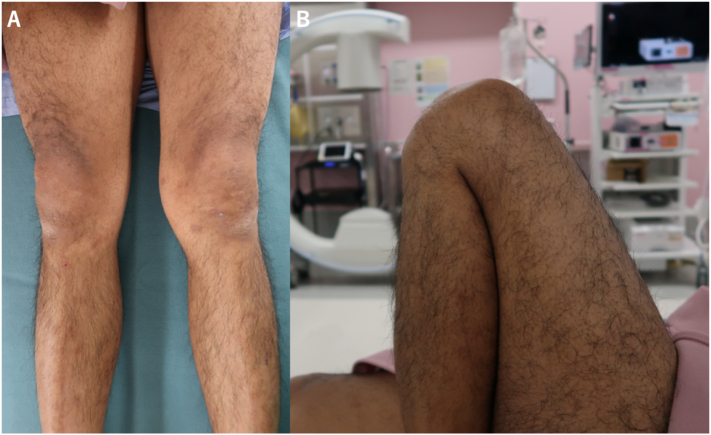
Preoperative clinical photograph. (A) The photograph showed that the left knee was valgus deformed. (B) There was no obvious flexion restriction.

On examination, the patient had an obvious valgus knee with 0°–140° of motion with no swelling, laxity, sagging, or neurovascular deficit (Fig. 1).

**Fig. 2:**
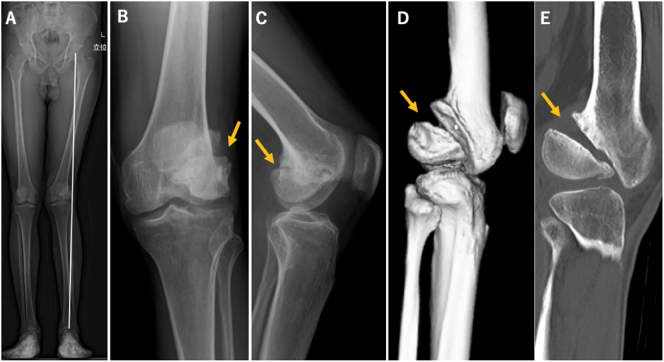
Conventional X-ray and computed tomography showed nonunion of the lateral Hoffa fracture. (A) Whole standing lower limb radiograph showed valgus deformity in standing position. (B) Anteroposterior view. (C) Lateral view. (D) 3D computed tomography (CT) reconstruction. (E) CT sagittal view.

Anteroposterior and lateral radiographs of the left knee revealed the nonunion of the left lateral Hoffa fracture (Letenneur type-III) [5] (Fig. 2). Magnetic resonance imaging showed the nonunion of the Hoffa fracture and lateral meniscus posterior tear on the other hand, but all other ligaments were intact. Lysholm Knee Scoring Scale was 48/100. As the patient had chronic knee pain due to the Hoffa fracture nonunion, he underwent open reduction and internal fixation.

### Surgical procedure

**Fig. 3:**
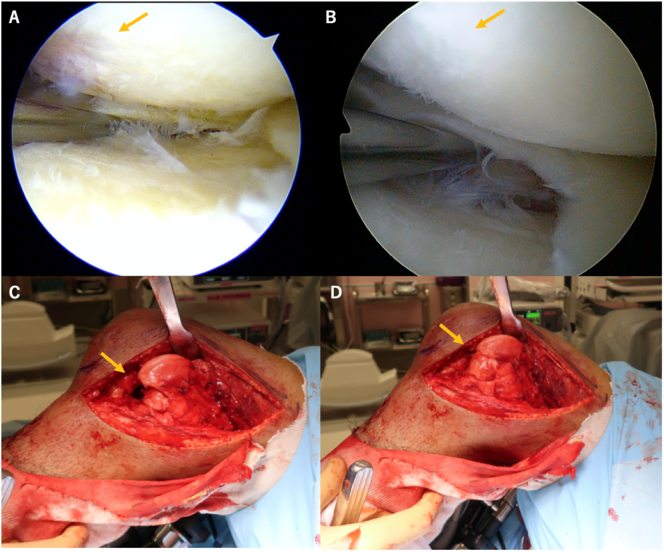
(A) Lateral meniscus posterior tear repair using all inside device. Overlying articular cartilage was partially damaged. (B) One year follow up, articular cartilage and lateral meniscus posterior tear were healed. Yellow arrow in (C) and (D) indicated that the posterior fragment was completely free and was displaced in the knee joint extension position and completely adjusted in the knee joint flexion position. (C) 45° of knee flexion. (D) 90° of knee flexion.

The patient was in a supine position, and the operative knee was held in the leg-drop position with 90° flexion with the patient under general anesthesia. Routine arthroscopic evaluation and lateral meniscus posterior tear repair using the all inside device [6] (Smith & Nephew Endoscopy, Andover, MA) were performed. Articular cartilage on the lateral femur was partially damaged (Fig. 3A). The knee joint was exposed using a lateral para-patellar approach to reach the nonunion site. Interposed fibrous tissue was resected and multiple drilling was made using 2-mm K-wire to refresh the both sides of nonunion sites. The posterior fragment was completely free; it was displaced in the knee joint extension position and completely adjusted in the knee joint flexion position (Fig. 3C–D). Bone grafting was not performed because of lack of bony defect due to Hoffa fracture. Anatomical reduction was confirmed by direct visualization of the articular surface. The fracture was fixed with three 4.5-mm headless screws and distal femoral locking plates (DepuySynthes, Solothurn, Switzerland).

### Postoperative management

**Fig. 4:**
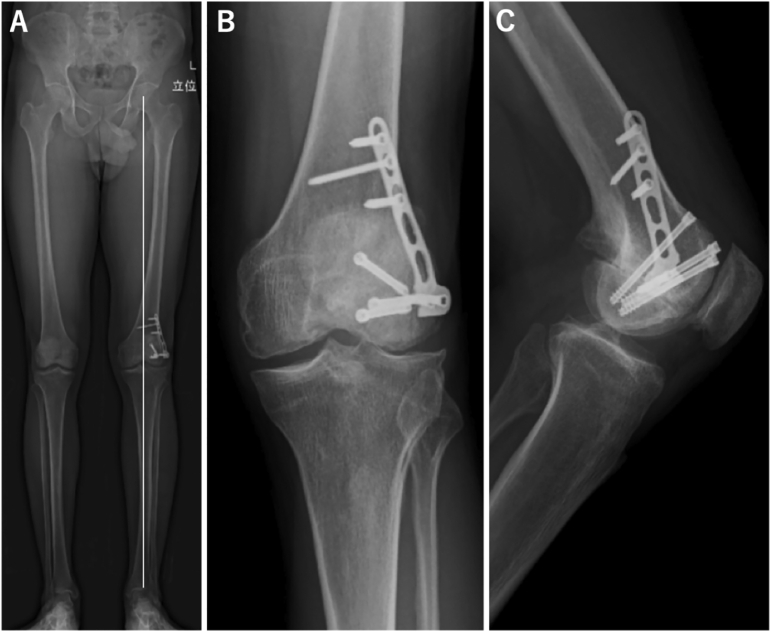
Follow up X-ray at 1 year. (A) Whole standing lower limb radiograph showed that the valgus deformity was corrected. (B) Anteroposterior view. (C) Lateral view.

Mobilization was started from the first operative day. One-third weight bearing was allowed 4 weeks postoperatively. Full weight bearing was allowed 8 weeks postoperatively. At the 1-year follow up, X-ray showed healing of the nonunion site with no displacement of the Hoffa fracture (Fig. 4). Second look arthroscopy revealed healed articular cartilage on the lateral femur (Fig. 3B). The knee range of the motion was 0° − 150°. Lysholm Knee Scoring Scale was improved to 90/100.

## Discussion

In the present case, internal fixation with LCP and HCS was performed for non-union of Hoffa fracture, and a good clinical course was achieved at 1 year postoperatively without bone grafting. There are few case reports describing the management of the nonunion of Hoffa fracture [7]. Cannulated cancellous screws and headless screws are most commonly used for Hoffa fractures [4]. However, only screw fixation is a weak construct, and it is difficult to contour recon or LCP plates according to the shape of the posterior femoral condyle [7]. Thus, we performed combined fixation of the headless screws and LCP plates to obtain better stabilization and allow early postoperative mobilization. Hoffa fractures are unstable due to the bony instability and the pull of the gastrocnemius and popliteus. Nonoperative management may lead to malunion or nonunion [3]. In contrast, Yuirui et al. found that a motionless pseudarthrosis was gradually formed by a proliferative osteophyte in the posteromedial aspect of the knee and provided sufficient support for knee motion [3]. In our case, the patient was treated nonoperatively and resulted in a nonunion with valgus deformity. Despite the chronicity, surgical intervention should be considered in the nonunion of Hoffa fractures.

No bone graft was performed in our case; Zhang et al. [1] described in their case report the need for cancellous bone graft for bridging of the epiphysis. Both of his cases were Letenneur type-II, whereas our case was Type III. Type-III Hoffa fractures have been shown to provide blood flow to the fracture fragment because of its attachment to the soft tissue. Therefore, we believe that good clinical results could have been obtained in this case without bone grafting.

## Conclusion

Nonunion of Hoffa fractures should be treated by an internal fixation despite the chronicity.

## Declaration of competing interest

The authors declare no conflict of interest.
